# Homozygous Loss of *Septin12*, but not its Haploinsufficiency, Leads to Male Infertility and Fertilization Failure

**DOI:** 10.3389/fcell.2022.850052

**Published:** 2022-04-25

**Authors:** Haixia Chen, Peng Li, Xiaoling Du, Yiding Zhao, Lingling Wang, Ye Tian, Xueru Song, Ling Shuai, Xiaohong Bai, Lingyi Chen

**Affiliations:** ^1^ Tianjin Key Laboratory of Female Reproductive Health and Eugenics, Reproductive Medicine Center, Department of Gynecology and Obstetrics, Tianjin Medical University General Hospital, Tianjin, China; ^2^ Tianjin Union Medical Center, State Key Laboratory of Medicinal Chemical Biology, Tianjin Key Laboratory of Protein Sciences, Frontiers Science Center for Cell Responses, National Demonstration Center for Experimental Biology Education and College of Life Sciences, Nankai University, Institute of Translational Medicine, Tianjin, China; ^3^ State Key Laboratory of Medicinal Chemical Biology and College of Pharmacy, Nankai University, Tianjin, China

**Keywords:** *SEPTIN12*, oocyte activation, fertilization failure, calcium oscillation, male infertility

## Abstract

The *SEPTIN12* gene has been associated with male infertility. Male *Septin12* ^+/−^ chimera mice were infertile, supporting the prevailing view that *SEPTIN12* haploinsufficiency causes male infertility. In this study, we identified a heterozygous mutation on *SEPTIN12*, c.72C>A (p.Cys24Ter) in the male partner of a patient couple, who had a previous fertilization failure (FF) after intracytoplasmic sperm injection (ICSI) and became pregnant after ICSI together with artificial oocyte activation (AOA). To investigate the role of *SEPTIN12* in FF and oocyte activation, we constructed *Septin12* knockout mice. Surprisingly, *Septin12* ^−/−^ male mice, but not *Septin12* ^+/−^ male mice, are infertile, and have reduced sperm counts and abnormal sperm morphology. Importantly, AOA treatment enhances the 2-cell embryo rate of ICSI embryos injected with *Septin12* ^*−/−*^ sperm, indicating that FF caused by male *Septin12* deficiency is overcome by AOA. Mechanistically, loss of PLCζ around the acrosome might be the reason for FF of *Septin12* ^*−/−*^ sperm. Taken together, our data indicated that homozygous knockout of *Septin12*, but not *Septin12* haploinsufficiency, leads to male infertility and FF.

## Introduction

Infertility is a worldwide health problem affecting about 15% reproductive-aged couples (Agarwal et al., 2015; Yeste et al., 2016). Assisted reproduction techniques (ARTs), especially intracytoplasmic sperm injection (ICSI), have allowed severe infertility couples to conceive. The procedure of ICSI is to inject a sperm into the oocyte cytoplasm, thus bypassing several key steps during fertilization, such as acrosome reaction, membrane fusion, and penetration into the oocyte. Even though the average fertilization rate in ICSI is around 70%, total fertilization failure still occurs in 1–3% of ICSI cycles (Van Steirteghem et al., 1993; Flaherty et al., 1998; Mahutte and Arici, 2003; Esfandiari et al., 2005). It has been revealed that more than 80% of unfertilized oocytes after ICSI are arrested at the metaphase II (MII), likely due to oocyte activation failure (OAF) (Flaherty S. P. et al., 1995; Flaherty S. et al., 1995; Flaherty et al., 1998). Thus, artificial oocyte activation (AOA), which triggers calcium oscillation by mechanical, electrical, or chemical stimuli, is applied to treat fertilization failure (FF) after ICSI. ICSI together with AOA (ICSI-AOA) improves reproductive outcomes in patients with previous FF (Sfontouris et al., 2015). Yet, not all patients benefit from AOA. Particularly, patients with oocyte-related activation deficiency show a less beneficial response to AOA treatment (Nasr-Esfahani et al., 2008; Vanden Meerschaut et al., 2012; Ferrer-Buitrago et al., 2018).

Obviously, identification of genetic defects responsible for FF allows us to accurately predict reproductive outcomes of AOA. PLCζ has been identified as a sperm oocyte-activating factor (Saleh et al., 2020). Injection of PLCζ mRNA into mouse oocytes activates calcium oscillation. Conversely, depletion of PLCζ in sperm extracts reduces their ability to induce calcium oscillation in oocytes (Cox et al., 2002; Saunders et al., 2002). The absence, abnormal localization, and genetic mutations of PLCζ in sperms have been identified in patients with low or total FF after ICSI (Yoon et al., 2008; Nomikos et al., 2011; Yelumalai et al., 2015; Escoffier et al., 2016; Nazarian et al., 2019; Torra-Massana et al., 2019; Dai et al., 2020; Mu et al., 2020; Yan et al., 2020; Yuan et al., 2020). It is highly possible that AOA treatment may efficiently improve reproductive outcomes for these patients. However, PLCζ defects are detected in only a subset of patients with low or total FF after ICSI, suggesting that additional genes involved in FF remain to be discovered.

*SEPTIN12* belongs to the SEPTIN family which are GTP-binding proteins with unique filament forming capabilities. It is expressed specifically in testis, and located in the neck and annulus regions of mature sperm (Shen et al., 2017). *SEPTIN12* forms complexes with other *SEPTINs*, 1, 2, 10 and 11 at the sperm neck, and 1, 4, 6 and 7 at the annulus, and is essential for the assembly of the connecting pieces and the annulus (Toure et al., 2011; Shen et al., 2017; Shen et al., 2020). It was first identified as a down-regulated gene in the testicular tissue of infertile men (Lin et al., 2006). Various mutations of *SEPTIN12* have been identified in infertile males, implying *SEPTIN12* as a male infertility gene (Kuo et al., 2012; Lin et al., 2012; Miyamoto et al., 2012; Geng et al., 2019; Rafaee et al., 2020). Moreover, it has been reported that male *Septin12* ^+/−^ chimera mice are infertile, indicating that haploinsufficiency of *Septin12* may lead to male infertility (Lin et al., 2009).

In this study, a couple with previous total FF after ICSI became pregnant after ICSI-AOA. A heterozygous mutation on *SEPTIN12*, c.72C>A; p.Cys24Ter, was identified in the male patient through whole exome sequencing. To study the function and mechanism of *SEPTIN12* in oocyte activation, *Septin12* knockout (KO) mice were generated. In contrast to previously reported infertile male *Septin12* ^+/−^ chimera mice (Lin et al., 2009), our male *Septin12* ^*+/−*^ mice are fertile. Only *Septin12* ^*−/−*^ male mice, with reduced sperm count and defective sperms, are infertile. The 2-cell embryo rate of ICSI embryos injected with *Septin12* ^*−/−*^ sperm increases from about 15 to 40% after AOA treatment. We further demonstrated that the acrosomal distribution of PLCζ is diminished in *Septin12* ^*−/−*^ spermatozoa, providing a possible explanation for why *Septin12* ^*−/−*^ sperm fails to activate oocyte after ICSI. In summary, complete loss of *Septin12*, but not haploinsufficiency of *Septin12*, causes FF and infertility. ICSI-AOA may overcome the FF caused by male *Septin12* deficiency.

## Materials and Methods

### Subjects

The female was 41-year-old and had been diagnosed with primary infertility for 3 years; BMI: 21.8 kg/m^2^; basal endocrine levels are normal; chromosome phenotype was 46XX. The male was 41 and diagnosed with oligospermia; BMI: 23.5 kg/m^2^, chromosome phenotype was 46XY; No Y-chromosome micro-deletions were detected; DNA fragment index (DFI) was 20.13%; high DNA stain-ability (HDS) was 18.38%.

### Intracytoplasmic Sperm Injection and Artificial Oocyte Activation

For human ICSI procedure, cumulus stripping was performed 2 h after oocyte retrieval to examine oocyte maturation. Denudation of cumulus cells was performed by the exposure of oocytes to HYASETM (Vitrolife, Sweden) for a maximum of 60 s. Denudation of cumulus cells was performed by the use of glass tube slightly. The oocytes were washed three times in G-MOPS-Plus (Vitrolife, Sweden) after denudation. Only Metaphase II (MII) oocytes were inseminated by ICSI using the partner’s spermatozoa.

Human ICSI-AOA was performed 4–6 h later after oocytes retrieval. Once ICSI finished, oocytes were incubated in culture medium for 10 min and then transfer to culture medium containing 10 μM calcium ionophore A23187 (Sigma, United States) for 10 min at 37°C and 6.0% CO_2_. The oocytes were then washed twice in culture medium and finally placed in culture medium (G-1 Plus, Vitrolife Sweden) in the incubator at 37°C and 6.0% CO2.

Mouse ICSI was performed as previously described with slight modification (Li et al., 2012). Briefly, mature oocytes at MII stage were collected from 8-week-old CD-1 female mice by hormone administration. Spermatids minced from the epididymis of C57 sex-matured male mice were decapitated, and prepared in M2 medium (Sigma, M7167) for the subsequent injection. For injection, sperm head suspension and oocytes were manipulated in M2 medium covered with mineral oil (Sigma, M8410). Each sperm head was injected into one oocyte with an 8-μm injection needle using a Piezo-drill equipment (Prime Tech, PMAS-C7150). Reconstructed embryos were further cultured in KSOM-AA medium (Millipore, MR-020P-5F) at 37°C, 5% CO_2_. For artificial oocyte activation, the reconstructed embryos were activated by 10 mM SrCl_2_ (Sigma, 255521) in calcium-free CZB medium (Shuai et al., 2014) supplemented with 5 μg/ml of cytochalasin B (MCE, HY-16928) at 37°C, 5% CO_2_ for 6 h. The activated embryos were transferred to KSOM-AA medium at 37°C, 5% CO_2_ for further culture. The development efficiency of embryos was calculated every day until blastocyst stage.

### Assessment of Fertilization and Embryo Transfer

Fertilization was scored 16–18 h after injection and insemination. Normal fertilization was when two pronuclei appear, abnormal fertilization when less or more than two pronuclei appear. The embryos were evaluated 24 h later (42 h after insemination or injection) by laboratory staff member. Embryos were cultured in the Vitrolife series of culture medium drops covered with mineral oil. Embryo transfer was performed on day 3 after injection. Luteal phase support with progesterone was administered from the day following oocyte retrieval until the day of the pregnancy test.

### Whole Exome Sequencing

Whole exome sequencing was performed by AEGICARE (Shenzhen, China). Briefly, genomic DNA was isolated from peripheral blood samples using DNA extraction kit. Exon sequences were enriched and subjected to DNA sequencing on Illumina HiSeq 2000. Sequencing depth reached 123×. Copy number variation was analyzed with the Weaver (AEGICARE, Shenzhen, China). The reference genome GRCh37 was used for sequence alignment. The sequencing data is available in sequence read archive (SRA, accession number PRJNA753965).

### Sanger Sequencing

The mutation site in the exon 2 of *SEPTIN12* was verified by PCR and Sanger sequencing. The DNA fragment was amplified with primers 5′-gca​gct​cct​gga​agc-3′ and 5′-ggc​tat​gaa​gat​ggg​gtt​t-3′.

### Generation of *Septin12* Knockout Mouse

The *Septin12* KO mice were constructed by Cyagen (Guangzhou, China). The sgRNAs targeting *Septin12* and Cas9 mRNA were co-injected into mouse zygotes to generate targeted KO offspring. The genotype was determined by PCR with the following primers. Forward primer: 5′- tca​gag​taa​ccc​ctc​tga​gcc-3′; reverse primer 1 (R1): 5′-att​taa​ttt​cag​ccc​tcc​tgt​gag-3′; reverse primer 2 (R2): 5′-gct​cta​tac​cta​acg​tcc​tgt​gg-3′. *Septin12* ^−/−^ mice were obtained by mating between *Septin12* ^+/−^ mice.

### Tissue Section and H & E Staining

The testis and epididymis were dissected immediately after euthanasia, fixed in 4% paraformaldehyde (PFA) for up to 24 h, dehydrated from 100% ethanol to 70% ethanol, and embedded in paraffin. The embedded tissues were cut into 5 μm sections, and mounted on glass slides. The sliced sections were deparaffinized, rehydrated, and stained with hematoxylin solution (Biosharp, BL702A) and 0.5% eosin (Solarbio, 15086-94-9) for histological examination.

### Immunofluorescence

For immunofluorescence assay of tissue sections, deparaffinizated sections described above were washed in phosphate-buffer saline (PBS) for 5 min. These sections were boiled in sodium citrate buffer for antigen retrieval for 15 min, and blocked in 5% BSA for 45 min. For immunofluorescence of spermatozoa, slides were treated with 3-Aminopropyl (APES, Sigma, 919-30-2) in advance. 50 μl of sperms at 1 × 10^6^/ml was added onto the APES treated slides. Slides were fixed in 4% PFA, and blocked in 5% BSA supplemented with 0.3% Triton X-100 for 30 min. Slides were incubated with primary antibody, anti-*SEPTIN12* (1:100, Abnova, H00124404-M), or anti-PLCζ (1:100, Abcam, ab124446), overnight at 4°C, and then detected by Alexa Fluor 488-AffiniPure Goat Anti-Mouse IgG (Jackson, 115-545-003) or Alexa Fluor 488-AffiniPure Goat Anti-Rabbit IgG (111-545-003). Hoechst 33342 was used for nucleus staining. Images were captured by Zeiss microscope with CCD.

For acrosome staining of tissue sections, after blocking in 5% BSA, slides were incubated with Alexa Fluor 488-lectin-PNA (20 μg/ml, Invitrogen, L21409) for 30 min, and then washed by PBS for three times. For acrosome staining of spermatozoa, slides were fixed by 4% PFA, permeabilized with 1% Triton X-100 for 5 min, incubated with 20 μg/ml Alexa Fluor 488-lectin-PNA at 37°C for 30 min, and then washed by PBS.

### Sperm Count and Motility Analysis

The cauda epididymis was dissected from adult mice. Sperms were extruded from the cauda epididymis and incubated for 15 min at 37°C in Human Tubal Fluid (HTF) medium. Hemocytometer was used for counting. For mouse motility analysis, a microscope (OLYMPUS, CX41) with the CASA system (Beijing Suijia) was used. At least 200 tracks were measured for each group.

### Calcium Oscillation Assay

Oocytes were prepared as described in mouse ICSI procedure. Mature oocytes were treated with 0.3 mg/ml hyaluronidase for 5 min at 37°C, and then washed twice with M2 medium. Oocytes were incubated in M2 drops supplemented with 5 μM Fluo-4 AM (Invitrogen, F14217) at 37°C for 30 min, and then washed twice with M2 medium. Oocytes were incubated in M2 drops at 37°C for 15 min to remove excess dye. At the same time, sperms were isolated from the epididymis as described in mouse ICSI procedure. After ICSI, embryos were transferred into a KSOM drop covered by mineral oil, and imaged with live cell imaging microscope (Leica, AF7000) every 30 s and continuously for 1 h.

### Statistical Analysis

All data are represented as means ± SD. Statistical differences were analyzed through Student’s t test. *p*-values were considered significant at *p < 0.05; **p < 0.01; ***p < 0.001.

## Results

### Intracytoplasmic Sperm Injection-Artificial Oocyte Activation Allowed Pregnancy in a Couple With Previous Fertilization Failure After Intracytoplasmic Sperm Injection

A couple, who had a failed ICSI attempt in another hospital, came to our reproductive medicine center for fertility treatment. The female was diagnosed with primary infertility for 3 years, and the male was diagnosed with oligospermia (Table 1). In the previous failed cycle, twenty MII oocytes were retrieved and then inseminated by ICSI. None of the twenty MII oocytes were fertilized. In our center, thirty-seven oocytes were obtained, among which thirty oocytes were at the MII stage. Considering the history of complete FF, thirty MII oocytes were randomly divided into two groups, and inseminated by conventional ICSI and ICSI-AOA respectively. In the ICSI group, only 1 out of 17 oocytes had two pronuclei, and this fertilized oocyte failed to develop to Day 5. In contrast, in the ICSI-AOA group, 12 out of 13 oocytes were fertilized, and 8 out of these 12 embryos developed to the 8-cell stage (Table 2). After two 8-cell embryos were transferred in uterus, the female patient was successfully pregnant and gave birth to a child.

**TABLE 1:** Semen assessment of the male patient.

Patient	Age	Sperm concentration	Total motility (%)	Progressive motility (%)	Sperm morphology
Male patient	41	15.1 × 10^6^/ml	38	19	Normal
		7.5 × 10^6^/ml	66	33	Normal

**TABLE 2:** The outcomes of ICSI and ICSI-AOA.

Patient and procedure	Year	No. of follicles	No. of mature oocytes injected	AOA (n)	No. of 2PN oocytes	No. of high-quality embryos
Clinic patient-ICSI	2017	20	20	-	0	0
Clinic patient-ICSI	2019	37	30 (Two treatments)	− (17)	1	0
				+ (13)	12	8

### Identification of a *SEPTIN12* Mutation in the Male Patient

To identify the genetic defect responsible for the OAF in the couple, whole exome sequencing was performed. No pathogenic copy number variation was detected in the exomes of the couple. We then looked for mutations on infertility genes. Four heterozygous mutations in *SEPTIN12*, *FSIP2*, *CFAP69*, and *CEP19*, in the male patient, and two heterozygous mutations in *MSH5* and *MCM8*, in the female patient, were identified (Table 3). Among these mutated infertility genes, mutations of *FSIP2*, *CFAP69*, *CEP19*, *MSH5* or *MCM8* are recessive for spermatogenic failure or premature ovarian insufficiency (Guo et al., 2017; Dong et al., 2018; Martinez et al., 2018; Yildiz Bolukbasi et al., 2018; He et al., 2019; Liu et al., 2019; Zhang et al., 2020). Thus, the heterozygous mutations of these five genes are unlikely to be the causative mutation for the OAF phenotype. In contrast, given that male *Septin12* ^+/−^ chimera mice are infertile (Lin et al., 2009), haploinsufficiency of *Septin12* was believed to cause male infertility. Therefore, we further investigated the role of *SEPTIN12* in OAF after ICSI.

**TABLE 3:** Mutations in infertility genes identified by whole exome sequencing.

Patient	Mutant gene	Chromosome location	Genomic mutation	Protein mutation	Mutation genotype	Disease associated with mutated gene (Mode of inheritance)
Male	*SEPT12*	Chr16:4837575	NM_14460 5.4: c.72C>A	Cys24Ter	Heterozygous	Spermatogenic failure 10 (AD)
	*FSIP2*	Chr2:186669325	NM_17365 1.3: c.15292A>G	Ile5098Val	Heterozygous	Spermatogenic failure 34 (AR)
	*CFAP69*	Chr7:89912209	NM_00103970 6.2: c.1376C>T	Pro459Leu	Heterozygous	Spermatogenic failure 24 (AR)
	*CEP19*	Chr3:196435431	NM_03289 8.4: c.110T>C	Ile37Thr	Heterozygous	Morbid obesity and spermatogenic failure (AR)
Female	*MSH5*	Chr6: 31726557	NM_17216 5.3: c.1231A>G	Met411Val	Heterozygous	Premature ovarian failure 13 (AR)
	*MCM8*	Chr20: 5939343	NM_03248 5.5: c.760C>T	Pro254Ser	Heterozygous	Premature ovarian failure 10 (AR)

AD: autosomal dominant; AR: autosomal recessive.

### *Septin12* ^*−/−*^ Male Mice are Infertile and Have Defective Spermatozoa

**FIGURE 1:**
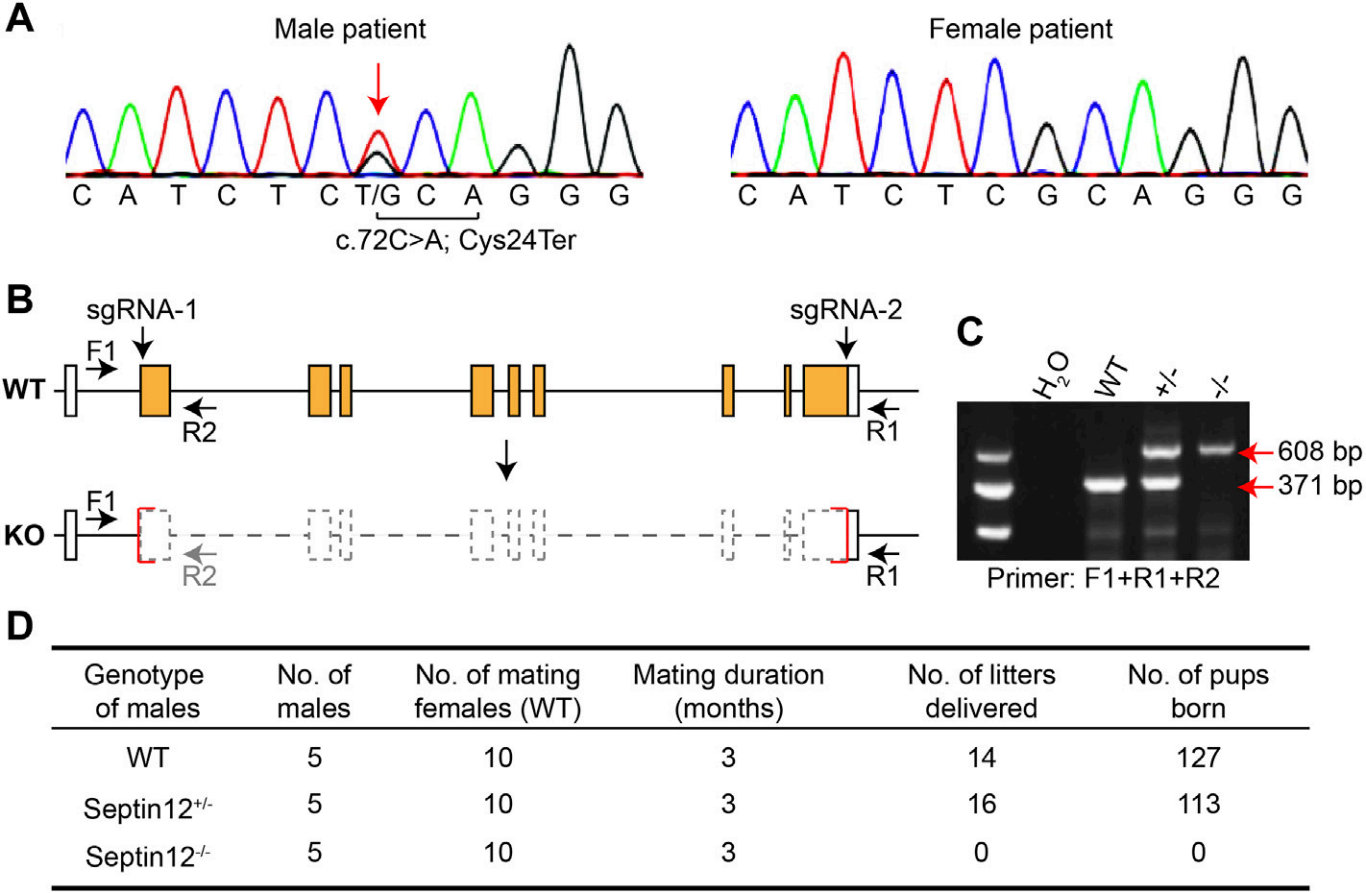
Male infertility of *Septin12* ^−/−^ mice. **(A)** Sequencing chromatograph showing the c.72C>A mutation on *SEPTIN12* in the male patient. **(B)** Schematic illustration of the strategy for knocking out the *Septin12* gene by CRISPR/Cas9. Genotyping primers F1, R1, and R2 are marked by arrows. **(C)** Genotyping of WT, *Septin12* ^+/−^, and *Septin12* ^−/−^ mice by PCR. A 371 bp band is amplified from the WT allele with primers F1 and R1, while a 608 bp band is amplified from the KO allele with primers F1 and R2. **(D)** Male fertility of WT, *Septin12* ^+/−^, and *Septin12* ^−/−^ mice.

We first validated the heterozygous variant on *SEPTIN12*, c.72C>A, identified in the male patient, by PCR and Sanger sequencing (Figure 1A). This variant changes the cysteine codon (TGC) at position 24 to a stop codon (TGA) (p.Cys24Ter), leading to the deletion of *SEPTIN12*.

**FIGURE 2:**
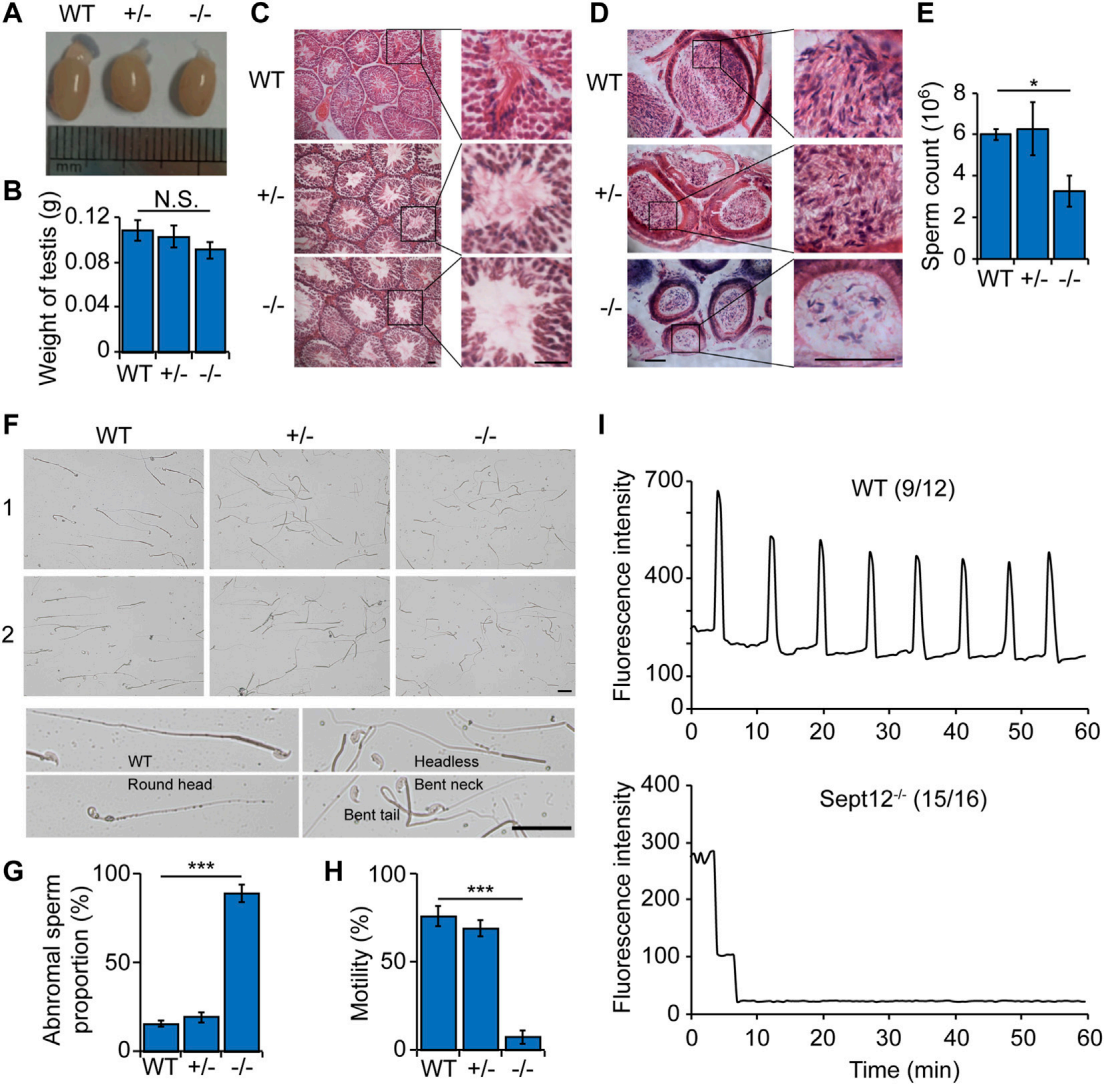
Abnormal sperm morphology and motility in *Septin12* ^−/−^ male mice. **(A)** The morphology of the testis from WT, *Septin12* ^+/−^, and *Septin12* ^−/−^ mice. **(B)** Testis weight of WT, *Septin12* ^+/−^, and *Septin12* ^−/−^ mice (*n* = 5 for each genotype). NS stands for not statistically significant. **(C,D)** Hematoxylin and eosin staining of testis **(C)** and epididymis **(D)** sections from WT, *Septin12* ^+/−^, and *Septin12* ^−/−^ mice. Scale bars, 100 μm. **(E–H)** Sperm count **(E)**, morphology **(F,G)**, and motility **(H)** of WT, *Septin12* ^+/−^, and *Septin12* ^−/−^ mice (*n* = 5 for each genotype). Sperms were isolated from the epididymis. The data are represented as means ± SD, ***p < 0.001. **(F)** Scale bars, 10 μm. **(I)** Ca^2+^ oscillation profiles of ICSI embryos with sperms from WT mice (left panel) or *Sept12* ^*−/−*^ mice (right panel). The numbers in parentheses denote the fraction of embryos presented in the plots. The numerator is the number of ICSI embryos represented in the plot from two independent experiments, and the denominator is the total number of ICSI embryos from two independent experiments.

To investigate the role of *SEPTIN12* in OAF, we established *Septin12* knockout mice using CRISPR/Cas9, and the genotypes of mice were determined by PCR (Figures 2B,C). Nearly the whole open reading frame of *Septin12* is deleted in the KO allele (Figure 2B), thus mimicking the deletion of *SEPTIN12* in the male patient. To our surprise, male *Septin12* ^+/−^ mice are fertile. However, no pups were born through the mating between *Septin12* ^−/−^ male and WT female mice, indicating male infertility of *Septin12* ^−/−^ mice (Figure 2D). These data are seemly conflicted with the infertility in the male patient with the heterozygous c.72C>A *SEPTIN12* mutation and male *Septin12* ^+/−^ chimeric mice (Lin et al., 2009). We further discuss this issue in the later sections.

Analysis of reproductive organs revealed no obvious difference in testis morphology and weight in WT, *Septin12* ^+/−^ and *Septin12* ^−/−^ males (Figures 2A,B). Histological analysis of tissue sections showed that there are less spermatozoa in *Septin12* ^−/−^ testis and epididymis, compared with their WT and *Septin12* ^+/−^ counterparts (Figures 2C,D). Consistently, the sperm count in *Septin12* ^−/−^ males is about half of those in WT and *Septin12* ^+/−^ male mice (Figure 2E). Moreover, a large fraction of *Septin12* ^−/−^ spermatozoa exhibit abnormal morphologies, such as round spermatid, headless, bent neck, and tail defects (Figures 2F,G), and lack mobility (Figure 2H). These data suggested that defects in spermatozoa, rather than abnormal reproductive organs, leads to male infertility of *Septin12* ^−/−^ mice.

### Artificial Oocyte Activation Enhances the 2-Cell Embryo Rate After Intracytoplasmic Sperm Injection With *Septin12* ^−/−^ Sperm

Given the clinical result that ICSI-AOA allowed the patient couple with male *SEPTIN12* deficiency to conceive, we next tested whether *Septin12* ^−/−^ sperms are defective in oocyte activation after ICSI, and whether the FF after ICSI with *Septin12* ^−/−^ sperm can be overcome by AOA. ICSI experiments were performed using sperms from WT, *Septin12* ^+/−^, and *Septin12* ^−/−^ mouse, and WT mouse oocytes. Oocytes injected with WT and *Septin12* ^+/−^ sperms developed to the 2-cell stage with high efficiencies, 89.8 and 69.0%, respectively. In contrast, only 16.3 and 13.0% of oocytes injected with *Septin12* ^−/−^ sperms developed to 2-cell embryos, in two independent ICSI experiments (Table 4). These data indicated that male *Septin12* deficiency causes FF after ICSI. AOA treatment significantly enhanced the 2-cell embryo rate in the ICSI group with *Septin12* ^−/−^ sperm, from ∼15 to ∼40%, while the 2-cell embryo rate in the ICSI group with *Septin12* ^+/−^ sperm was only slightly increased by AOA (Table 4). These data validated that AOA treatment indeed overcomes the FF after ICSI caused by male *Septin12* deficiency. We further demonstrated that calcium oscillation is not properly triggered in ICSI embryos with *Septin12* ^−/−^ sperm (Figure 2I), indicating that failure in initiating calcium oscillation contributes to the FF after ICSI with *Septin12* ^−/−^ sperms.

**TABLE 4:** ICSI outcomes with sperms from WT, *Septin12* ^+/−^ and *Septin12* ^−/−^ mice.

Group	♀	♂	AOA	No. of reconstructed	2-Cell (%)	4-Cell (%)	Morula (%)	Blastocyst (%)
Control	WT	WT	−	49	44 (89.8)	38 (77.5)	29 (59.2)	23 (46.9)
I	WT	*Septin12* ^+/−^	−	58	40 (69.0)	22 (37.9)	16 (27.6)	5 (8.6)
			+	47	36 (76.6)	22 (46.8)	14 (29.8)	5 (10.6)
II-1	WT	*Septin12* ^−/−^	−	49	8 (16.3)	7 (14.3)	3 (5.9)	1 (2.0)
			+	51	20 (39.2)	19 (37.3)	9 (18.3)	2 (4.1)
II-2	WT	*Septin12* ^−/−^	−	23	3 (13.0)	1 (4.3)	0 (0.0)	0 (0.0)
			+	22	9 (40.1)	3 (13.6)	2 (9.1)	0 (0.0)

ICSI embryos with *Septin12* ^+/−^ sperm have lower developmental rates to various embryo stages, compared with ICSI embryos with WT sperm (Table 4). It implies that haploinsufficiency of *Septin12* might affect the quality of sperms. The slightly compromised quality of *Septin12* ^+/−^ sperms might be manifested in sub-optimal conditions, such as ICSI and *in vitro* embryo development, but not in *in vivo* fertilization and embryogenesis. It is obvious that the quality of *Septin12* ^−/−^ sperms are more severely impaired. Even with AOA treatment, only 2 out of total 73 embryos injected with *Septin12* ^−/−^ sperms developed to the blastocyst stage (Table 4), suggesting that defects in *Septin12* ^−/−^ sperms affect not only oocyte activation, but also the developmental potential of zygotes beyond the 2-cell stage.

### No Acrosomal Enrichment of PLCζ in *Septin12* ^−/−^ Sperms

**FIGURE 3:**
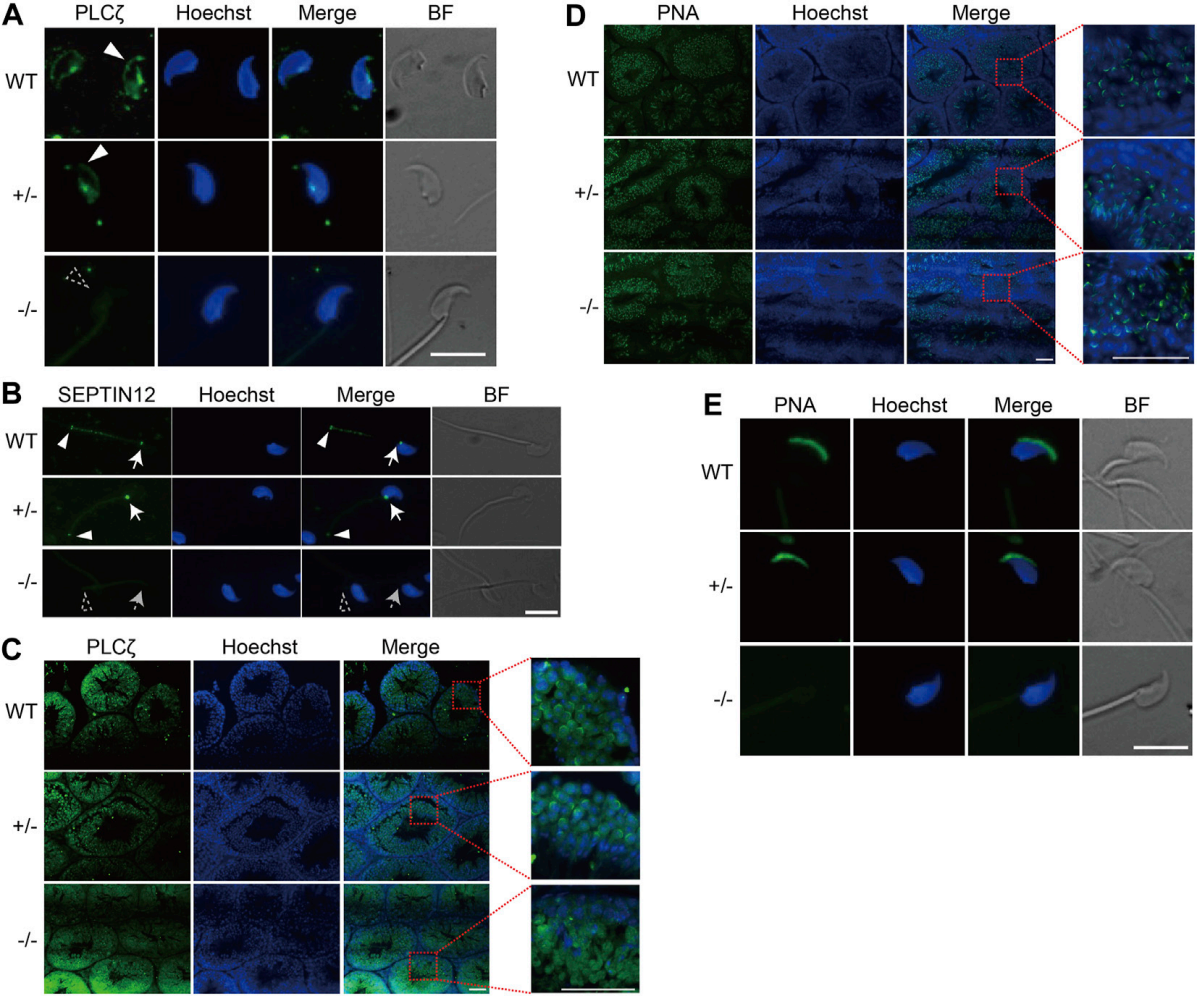
Disturbed distribution of PLCζ and acrosome malformation in *Septin12* ^−/−^ spermatozoa. **(A,B)** Immunofluorescence staining of PLCζ **(A)** and SEPTIN12 **(B)** in spermatozoa isolated from the epididymis of WT, *Septin12* ^+/−^, and *Septin12* ^−/−^ mice. Scale bars: 10 μm. **(A)** The PLCζ signal at the acrosome region is marked by solid white triangle, and the dashed triangle shows the lack of PLCζ signal at the acrosome region of *Septin12* ^−/−^ spermatozoa. **(B)** The signals of SEPTIN12 at the neck and annulus are indicated by solid white arrows and triangles, respectively. Dashed arrow and triangle point to the neck and annulus without SEPTIN12 signal. **(C,D)** Immunofluorescence staining of PLCζ **(C)** and acrosome **(D)** in testis sections of WT, *Septin12* ^+/−^, and *Septin12* ^−/−^ mice. Enlarge images are shown in the right side. Scale bars: 50 μm. **(D)** The acrosome was stained by Alexa Fluor 488-lectin-PNA. **(E)** Immunofluorescence staining of acrosome in spermatozoa isolated from the epididymis of WT, *Septin12* ^+/−^, and *Septin12* ^−/−^ mice. Scale bar: 10 μm.

We then addressed why *Septin12* ^−/−^ sperm fails to activate oocyte after ICSI. Since AOA treatment activates calcium oscillation and rescues the FF after ICSI due to male *Septin12* deficiency, it is very likely that *Septin12* ^−/−^ sperms are defective in triggering calcium oscillation upon injected into an oocyte. Sperm-specific PLCζ plays an essential role in inducing calcium oscillation (Cox et al., 2002; Saunders et al., 2002). Thus, immunofluorescence experiments were performed and revealed that the expression of PLCζ at the acrosomal and post-acrosomal regions is diminished in *Septin12* ^−/−^ spermatozoa isolated from cauda epididymis (Figure 3A), which might account for the FF after ICSI with *Septin12* ^−/−^ spermatozoa.

How does *SEPTIN12*, localized at the neck and annulus of spermatozoa (Figure 3B), affect the expression and distribution of PLCζ in the head of spermatozoa? It has been shown that during human spermiogenesis, *SEPTIN12* is first concentrated around the acrosome, and translocated to the neck and annulus regions in spermatozoa (Lin et al., 2011), implying that *SEPTIN12* might regulate the acrosomal distribution of PLCζ at the early steps of spermiogenesis. Indeed, immunofluorescent staining of testicular tissue sections revealed that PLCζ is enriched in the acrosome region of WT and *Septin12* ^+/−^ spermatids. In contrast, the acrosomal enrichment of PLCζ is diminished in *Septin12* ^−/−^ spermatids (Figure 3C). The acrosome formation appears to be normal in *Septin12* ^−/−^ spermatids (Figure 3D), suggesting that the disturbed distribution of PLCζ in *Septin12* ^−/−^ spermatids is not due to abnormal acrosome formation. Surprisingly, no acrosome signal in *Septin12* ^−/−^ spermatozoa isolated from cauda epididymis was detected by peanut agglutinin (PNA) staining (Figure 3E), reflecting no acrosome or lack of β-D-galactosylation on acrosomal membrane proteins. Given that the acrosome is formed normally in *Septin12* ^−/−^ testis, it is more likely that β-D-galactosylation of acrosomal membrane proteins is removed during sperm transit in the epididymis. Taken together, *Septin12* deficiency affects the acrosomal enrichment of PLCζ at the late cap phase and the early acrosome phase of spermiogenesis, and the formation of acrosome at the maturation phase.

## Discussion

AOA treatment improves reproductive outcomes in some patients, but not all, with previous FF after ICSI. Thus, identification of genetic defects responsible for FF which may be overcome by AOA is important for selective application of ICSI-AOA in patients with high beneficial potential. In this study, we showed that *Septin12* ^−/−^ male mice, but not *Septin12* ^+/−^ male mice, are infertile. Importantly, the 2-cell embryo rate of ICSI embryos with sperms from *Septin12* ^−/−^ mice was increased by AOA treatment (Table 4). These data suggest an essential role of *SEPTIN12* in oocyte activation and male infertility.

The male infertility in *Septin12* ^−/−^ mice seems to be conflicted with the infertile phenotype in the male patient with a heterozygous c.72C>A *SEPTIN12* mutation. Compound mutation might account for the infertility in the male patient. Consistently, in addition to the *SEPTIN12* mutation, heterozygous mutations were identified in three other infertility genes in the male patient (Table 3). Further studies are necessary to address whether compound mutations of *SEPTIN12* and other infertility genes lead to infertility. Nevertheless, we cannot rule out the possibility that heterozygosity for the null *SEPTIN12* mutation leads to male infertility in the human, but not in the mouse, due to species difference.

Our study also clarified the role of *SEPTIN12* in mouse spermatogenesis. Previous studies showed that male *Septin12* ^+/−^ chimeric mice are infertile (Lin et al., 2009). However, our male *Septin12* ^+/−^ mice are fertile, whereas male *Septin12* ^−/−^ mice are sterile. The main difference is that their *Septin12* ^+/−^ chimeric mice were generated by blastocyst injection of *Septin12* ^+/−^ embryonic stem cells (ESCs), while our *Septin12* ^+/−^ founder mice were established by CRISPR/Cas9 mediated gene editing in the zygote. The quality of injected *Septin12* ^+/−^ ESCs might affect the experimental result. For example, these ESCs might harbor a mutation in addition to the knockout of *Septin12*, thus impairing the development and maturation of sperms. The genetic background difference might also contribute to the conflicted results. The *Septin12* ^+/−^ ESCs were derived from the 129Sv mouse (Lin et al., 2009), while our *Septin12* knockout mice are in the C57BL/6 background. With current data, it is convincing that male *Septin12* ^+/−^ C57BL/6 mice are fertile.

*Septin12* ^−/−^ mouse spermatozoa display multiple defective phenotypes, including round spermatid, headless, bent neck, tail defects, and reduced mobility. These phenotypes are directly associated with the function of *SEPTIN12* in the neck and annulus regions of spermatozoa. ICSI should be able to treat the male infertility caused by these defects. In addition, *Septin12* ^−/−^ mouse spermatozoa have other defects not directly related to the neck and annulus regions, such as abnormal PLCζ distribution and acrosome formation. These defects reflect the function of *SEPTIN12* out of the neck and annulus regions. During spermiogenesis, *SEPTIN12* migrates from the acrosome to the neck and the annulus (Lin et al., 2011). Its presence in the acrosomal region appears to be essential for the acrosomal recruitment and enrichment of PLCζ during the late cap and the early acrosome phases, as well as the acrosome formation at the maturation phase. The acrosome defect should be overcome by ICSI, while the abnormal PLCζ distribution might lead to OAF, and thus require the treatment of ICSI-AOA. ICSI-AOA leads to high fertilization and pregnancy rate for globozoospermia patients (Tavalaee et al., 2018; Modarres et al., 2019). However, it is notable that even with ICSI-AOA, low blastocyst rates, 10.6 and 0–4.1%, were achieved for embryos injected with *Septin12* ^+/−^ and *Septin12* ^−/−^ sperms, respectively (Table 4). It implies that *Septin12* deficiency may cause additional defect(s) in sperms, which compromise the *in vitro* developmental potential of zygotes, and are not rescued by ICSI-AOA. Nuclear defects have been observed in sperms isolated from *Septin12* ^+/−^ chimeric mice (Lin et al., 2011). Consistently, DNA fragment index (DFI) and high DNA stain-ability (HDS) of the male patient were 20.13 and 18.38%, respectively, indicating DNA defects in the spermatozoa. Further investigations are required to characterize these additional defects in *Septin12* ^−/−^ sperms.

## Data Availability

The datasets presented in this study can be found in online repositories. The names of the repository/repositories and accession number(s) can be found below: https://www.ncbi.nlm.nih.gov/, PRJNA753965.
